# The existence of C_4_-bundle-sheath-like photosynthesis in the mid-vein of C_3_ rice

**DOI:** 10.1186/s12284-016-0094-5

**Published:** 2016-05-10

**Authors:** Weijun Shen, Luhuan Ye, Jing Ma, Zhongyuan Yuan, Baogang Zheng, Chuangen LV, Ziqiang Zhu, Xiang Chen, Zhiping Gao, Guoxiang Chen

**Affiliations:** College of Life Sciences, Nanjing Normal University, 1 Wenyuan Road, Nanjing, 210023 China; Institute of Food and Crops, Jiangsu Academy of Agricultural Sciences, 50 Zhongling Street, Nanjing, 210014 China; University of Illinois at Urbana-Champaign, Urbana, IL 61801 USA

**Keywords:** C_3_ and C_4_ photosynthesis, Cyclic/linear electron flow, Dysfunctional PSII, Mid-vein, Rice

## Abstract

**Background:**

Recent studies have shown that C_4_-like photosynthetic pathways partly reside in photosynthetic cells surrounding the vascular system of C_3_ dicots. However, it is still unclear whether this is the case in C_3_ monocots, especially at the molecular level.

**Results:**

In order to fill this gap, we investigated several characteristics required for C_4_ photosynthesis, including C_4_ pathway enzymes, cyclic/non-cyclic photophosphorylation rates, the levels and assembly state of photosynthetic machineries, in the mid-veins of C_3_ monocots rice with leaf laminae used as controls. The signature of photosystem photochemistry was also recorded via non-invasive chlorophyll a fluorescence and reflectance changes at 820 nm in vivo. Our results showed that rice mid-veins were photosynthetically active with higher levels of three C_4_ decarboxylases. Meanwhile, the linear electron transport chain was blocked in mid-veins due to the selective loss of dysfunctional photosystem II subunits. However, photosystem I was sufficient to support cyclic electron flow in mid-veins, reminiscent of the bundle sheath in C_4_ plants.

**Conclusions:**

The photosynthetic attributes required for C_4_ photosynthesis were identified for the first time in the monocotyledon model crop rice, suggesting that this is likely a general innate characteristic of C_3_ plants which might be preconditioned for the C_4_ pathway evolution. Understanding these attributes would provide a base for improved strategies for engineering C_4_ photosynthetic pathways into rice.

**Electronic supplementary material:**

The online version of this article (doi:10.1186/s12284-016-0094-5) contains supplementary material, which is available to authorized users.

## Background

C_4_ plants partition photosynthetic reactions between two distinct cell types: vascular bundle sheath (BS) and mesophyll (M) cells, a structure called ‘Kranz’ anatomy (Majeran and van Wijk 2009). Atmospheric CO_2_ is firstly fixed as C_4_ acids by phosphoenolpyruvate carboxylase (PEPC) in M cells, then the C_4_ acids are transferred into BS cells to be degraded by decarboxylating enzymes. The released CO_2_ is refixed by ribulose-1, 5-bisphosphate carboxylase/oxygenase (RuBisCO) and incorporated into the C_3_ cycle (Furbank 2011). This structural specialization of leaf tissue allows optimum operation of the C_4_ photosynthetic pathway to concentrate CO_2_.

C_4_ plants are divided into three biochemical subtypes based on different decarboxylating mechanisms: nicotinamide adenine dinucleotide phosphate-dependent malic enzyme (NADP-ME), nicotinamide adenine dinucleotide-dependent malic enzyme (NAD-ME), and phosphoenolpyruvate carboxykinase (PEPCK) types (Yoshimura et al. 2004). The majority of C_4_ crop species belong to the NADP-ME group (Furbank 2011) in which BS cells utilize malate as the C_4_ acid. Since malate decarboxylation results in a donation of reductive power, the reduced form of nicotinamide adenine dinucleotide phosphate (NADPH), substantial production of additional ATP would be required in BS cells (Finazzi et al. 2002). Therefore, NADP-ME C_4_ monocots such as sorghum (*Sorghum bicolor*) have completely agranular BS. They lack linear electron flow for NADPH generation due to photosystem II (PSII) deficiency and only function to generate ATP by photosystem I (PSI)-mediated cyclic electron flow (Voznesenskaya et al. 1999). The suppression of PSII activity mainly results from selective losses of subunits PsbP and PsbQ of the oxygen evolving complex (OEC) as well as PsbR, rather than the entire PSII complex (Meierhoff and Westhoff 1993).

It has been reported that a C_4_-like pathway existed in photosynthetic cells surrounding the vascular system (PCSVS) of C_3_ dicots tobacco and celery (Hibberd and Quick 2002). PCSVS of C_3_ plants is a more spatially separated version of the C_4_ photosynthetic pathway with high activity levels of three C_4_ photosynthesis decarboxylases, allowing to decarboxylate malate from the transpiration stream. This is also the case in mid-veins of *Arabidopsis*, a model C_3_ dicot (Brown et al. 2010). These findings suggest that essential biochemical components and the regulatory elements controlling the cell-specific gene expression required for C_4_ photosynthesis are already present in C_3_ plants. Thus, C_4_ photosynthesis can evolve from C_3_ plants with some modifications (Hibberd and Quick 2002).

Furthermore, PCSVS internal microenvironment and metabolic demands are similar to BS cells in NADP-ME C_4_ plants rather than C_3_ leaves. Because of fewer stomata and intercellular air spaces, but more layers of surrounding cells, the vascular tissue has been previously noted to reduce solubility and diffusivity of oxygen (Raven 1991; Hibberd and Quick 2002). Those further suppress mitochondrial respiration and result in ATP losses (Geigenberger 2003) in conjunction with the NADP^+^ requirement for high C_4_-acid decarboxylating activity (Yiotis et al. 2009). Therefore, the compensatory metabolic demands of higher ATP/NADPH ratios in mid-veins are comparable to that found in BS cells of NADP-ME type C_4_ plants.

These properties may require qualitative and quantitative adjustments of the photosynthetic attributes in such organs for both light and biochemical reactions (Yiotis and Manetas 2010). As observed in NADP-ME type C_4_ plants, chloroplasts in PCSVS of C_3_ plants deficient in linear but sufficient in cyclic electron flow would act as electron valves restoring the ATP/NADPH ratio (Yiotis et al. 2009). For example, Kotakis et al. (2006) show that twigs of *Eleagnus angustifolius* display low dark-adapted PSII photochemical efficiency and linear electron transport rates. Kalachanis and Manetas (2010) further demonstrate that the innately low linear flow is limited in the donor side (OEC) of PSII and the acceptor side of both PSII and PSI.

The origin, function and selective advantages of PCSVS in C_3_ lineages are critical for the understanding of the environmental, molecular and phylogenetic determinants for C_4_ evolution (Griffiths et al. 2012). With no doubt, more studies of PCSVS in C_3_ species are required to fill this fundamental gap (Leegood 2008) with molecular evidence. In particular, the neglected investigation on the monocot model plant rice (*Oryza sativa*), may have more significant impacts in Asia, because it would potentially simplify the approaches to generate a two-celled C_4_ shuttle in rice by expressing the classical enzymes of the NADP-ME C_4_ cycle (Kajala et al. 2011).

Following up the preliminary findings as mentioned above, we took advantage of the *O. sativa*. cv. Liangyoupeijiu as the plant material, which had large mid-veins. We have elucidated a wealth of photosynthetic traits of leaf laminae in the super hybrid rice (Zhang et al. 2007). In order to examine whether rice mid-veins had innate properties of C_4_-like photosynthesis, physiological traits, biochemical parameters, and spectral indicators were compared between leaf laminae and mid-veins in *O. sativa*.

## Results

### Rice mid-veins had photosynthesizable chloroplasts

**Fig. 1:**
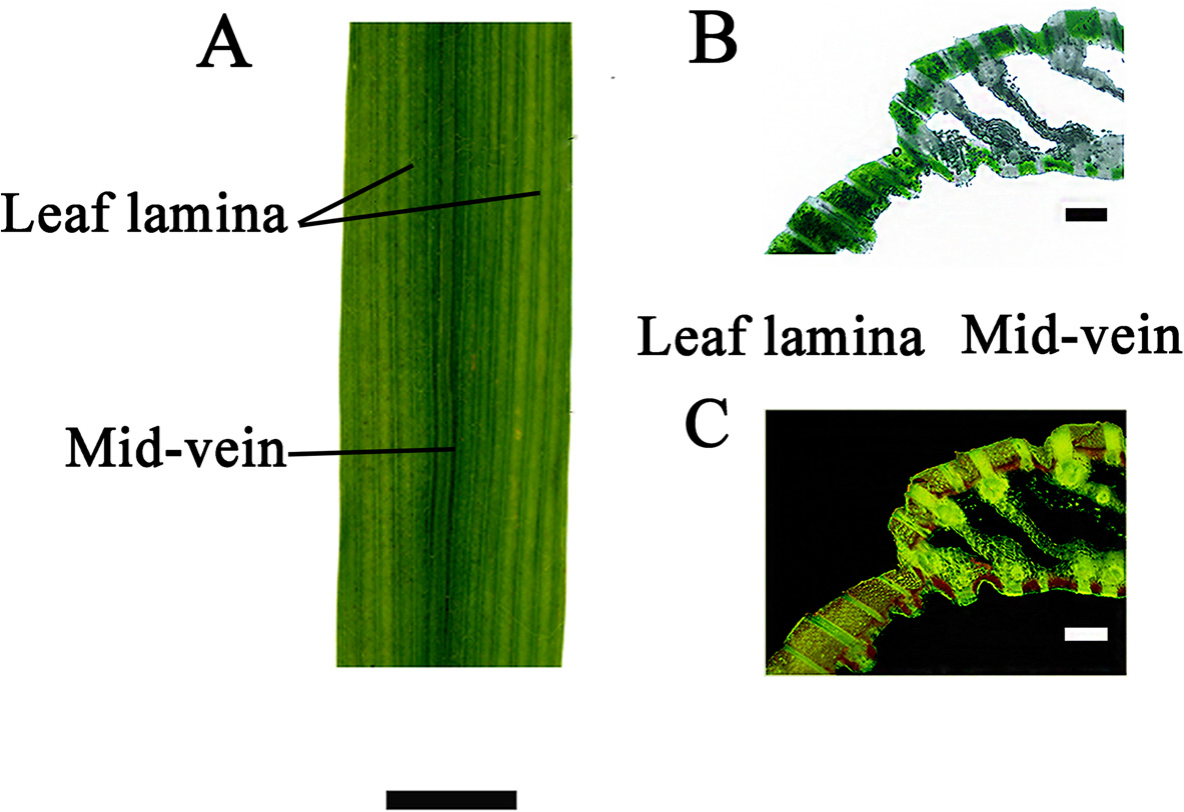
Phenotypic characteristics of rice mid-veins and leaf laminae. **a** The photograph of the midsection of a rice leaf, scale bar = 3 cm. **b** The optical micrograph of transverse sections of a rice leaf, and green areas illustrate the distribution of chlorophyllous cells. **c** The epifluorescence micrograph of transverse sections of a rice leaf, and chlorophyllous cells can be identified by the bright red fluorescence, scale bar =250 μm. Transverse sections of leaves were subjected to the epifluorescence microscopy. However, because of the intrinsic background noise from the epifluorescence microscopy and the low resolution of the Insight camera (CFI60, Nikon), images had to be first treated with Photoshop (CS5, Adobe) to discard the noise, which may have affected their quality (Wittmann et al. 2006; Berveiller and Damesin 2008)

The presence of chlorophyll (Chl) in chloroplasts caused greenness and bright red fluorescence under optical and epifluorescence microscopy, respectively (Berveiller and Damesin 2007). To examine the distribution of chlorophyllous cell in rice leaves, transverse sections of leaves were subjected to the microscopy (Fig. 1b and c). Our results demonstrated that both mid-veins and leaf laminae showed green regions under optical microscopy (Fig. 1b). Under epifluorescence microscopy, the red fluorescence signals emitted by the Chl also surrounded the xylem vessels in mid-veins (Fig. 1c).

### Rice mid-veins accumulated high levels of C_4_ acid decarboxylases

**Fig. 2:**
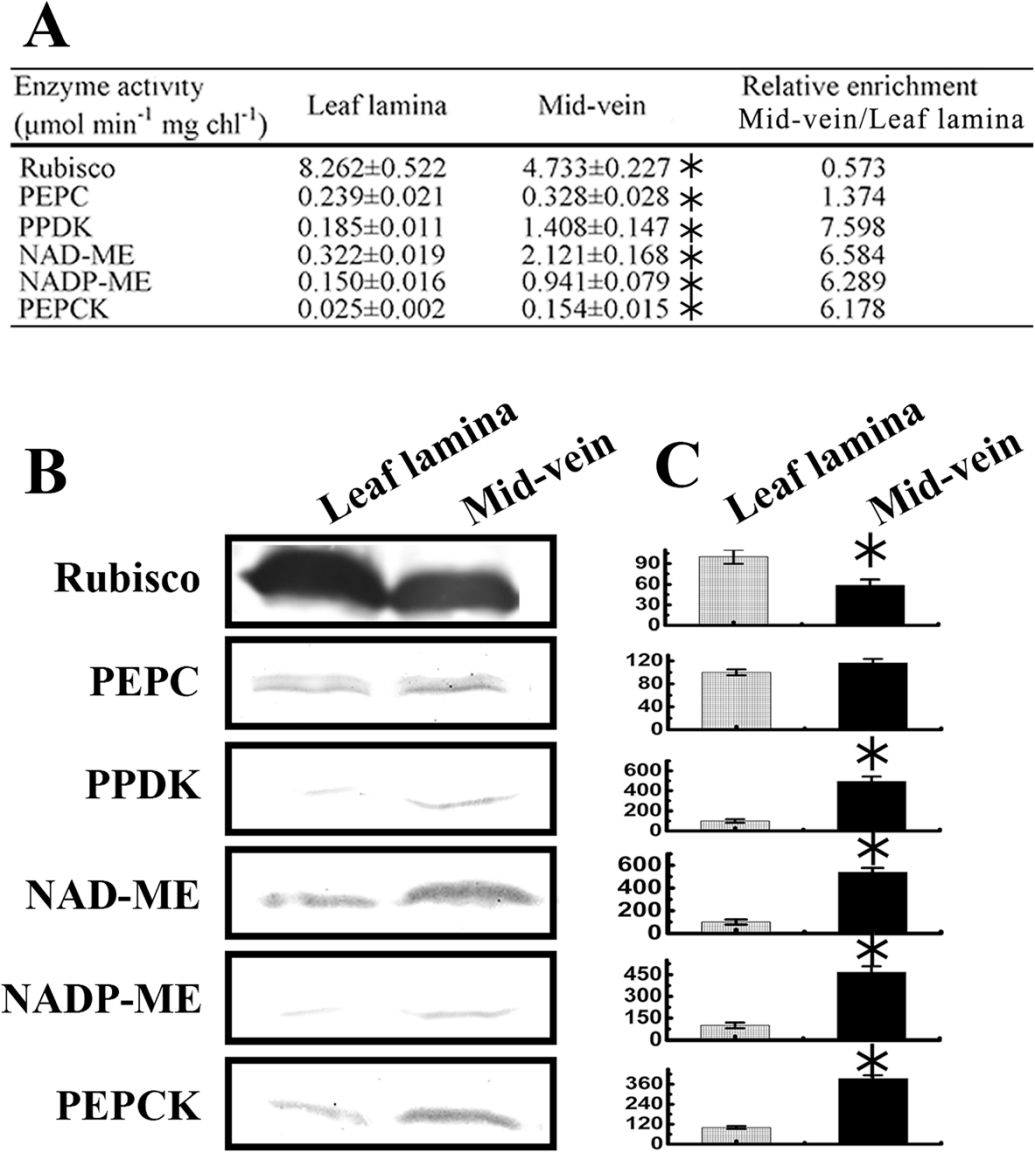
Enzymatic activities and protein levels in C_3_ or C_4_ cycle of mid-veins and leaf laminae. **a** Enzymatic activities are expressed as micromole per minute per mg Chl. **b** Immunoblot of C_3_ and C_4_ cycle enzymes (15 μg protein per spot). **c** Quantification of immunoblot data. Images were analyzed using Quantity One (Bio-Rad, USA). Data are means ± SE (*n* = 3), with the asterisk indicating statistically significant differences (*P* < 0.05) between mid-veins and leaf laminae

To test whether rice mid-veins possessed some enzymatic features of C_4_ BS cells or not, we evaluated various key enzymatic activities in either C_3_ or C_4_ cycles. The key enzyme of C_4_ cycle, PEPC, exhibited a little higher activity in rice mid-veins than in leaf laminae, while the key enzyme of C_3_ cycle, RuBisCO, had lower activity. It was worth noting that mid-veins showed greatly elevated activities for three C_4_ acid decarboxylases (NADP-ME, NAD-ME and PEPCK) and pyruvate phosphate dikinase (PPDK) per Chl unit: 6.2 to 7.6-fold greater than that of the leaf laminae (Fig. 2a). Immunoblot results showed that the enzyme protein levels were consistent with the enzymatic data (Fig. 2b). Mid-veins enriched more than 3.5-fold three C_4_ acid decarboxylases than leaf laminae, while the large subunit of RuBisCO was reduced by 42 % in mid-veins (Fig. 2c).

### Rice mid-veins showed unusual fluorescence signature of photosynthetic machineries

Fast Chl a fluorescence transients (F_t_) can provide information for the whole photosynthetic process from water splitting to PSI electron acceptor (feredoxin and NADP^+^) (Strasser et al. 2010). To study whether mid-veins operated unique photosynthetic machineries, fluorescence analysis was employed.

**Fig. 3:**
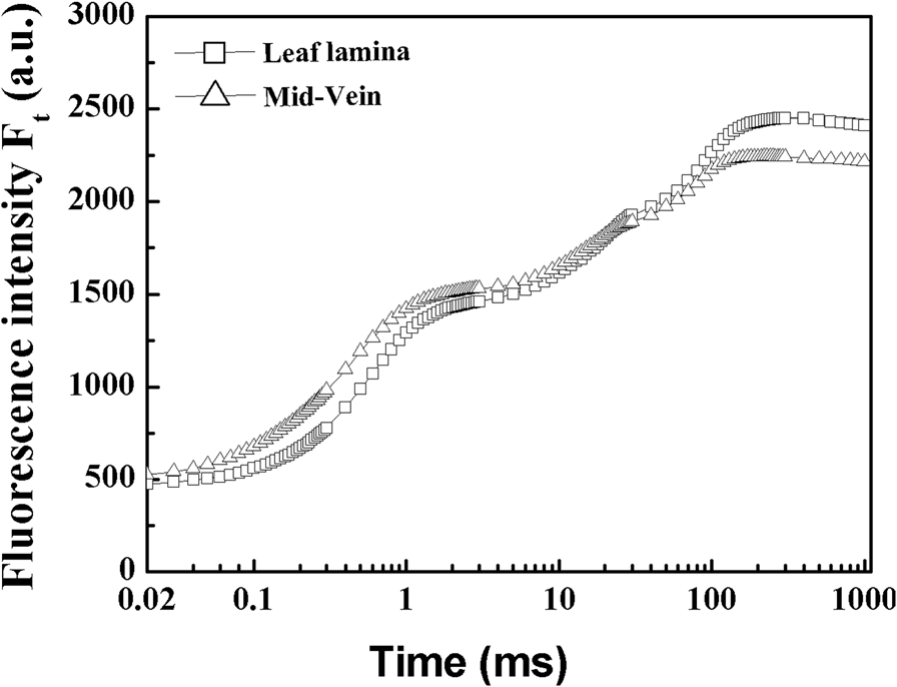
Fast Chl fluorescence kinetics (F_t_) of rice mid-veins (△) and leaf laminae (□). Each curve represents the average kinetics recorded from 15 independent measurements of 15 individual plants

As shown in Fig. 3, both leaf laminae and mid-veins displayed a typical and distinct polyphasic F_t_ rise, which meant that they were both photosynthetically active and not interfered by rectangular window fitted on the clip.

**Fig. 4:**
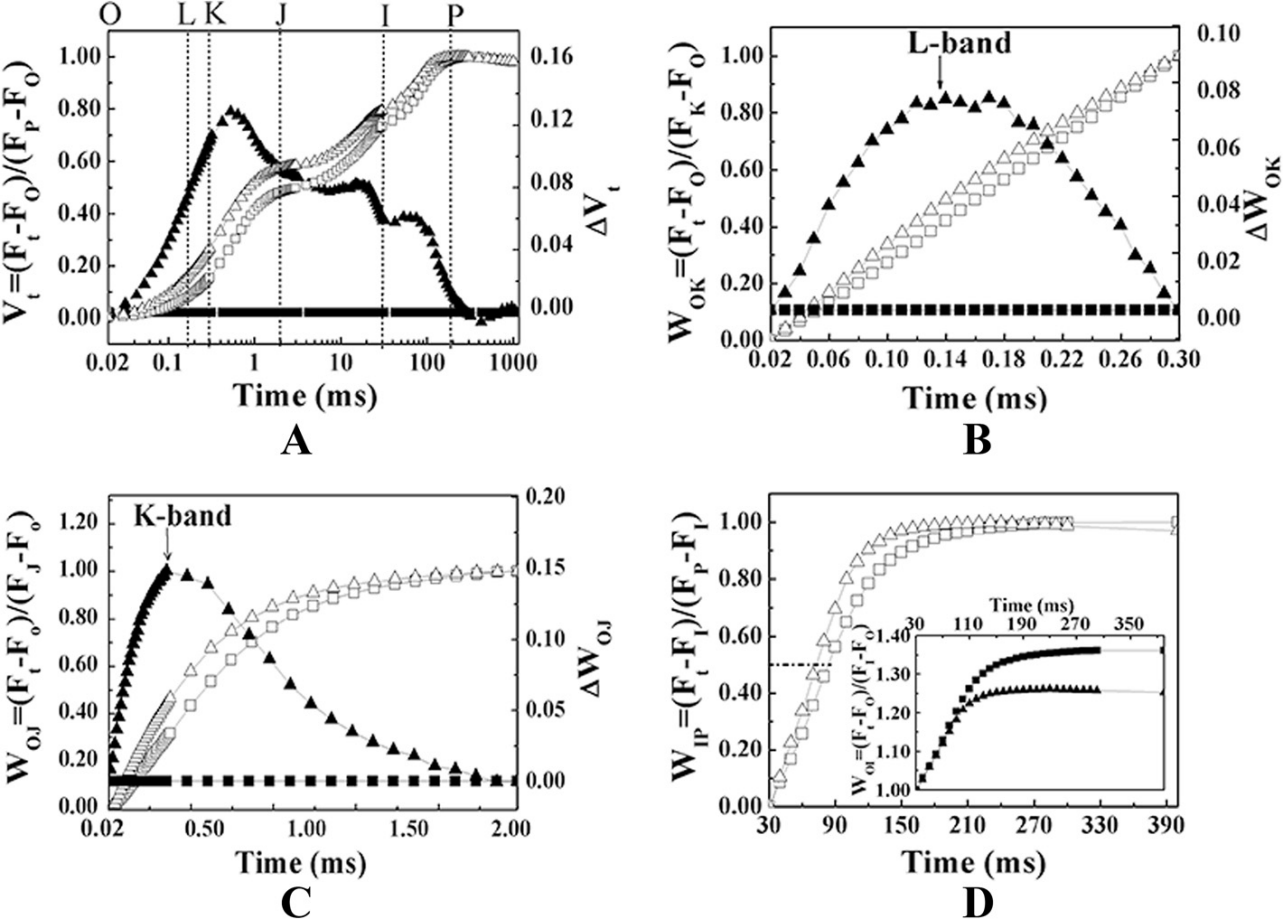
The different expressions of relative variable fluorescence (V or W, left vertical axis) of rice mid-veins (△) and leaf laminae (□). The difference fluorescence kinetics of mid-veins (▲) to leaf laminae (█) was calculated using the equation: ΔV (or ΔW) = V (or W)_mid ‐ vein_ – V (or W)_leaf laminae_ (right vertical axis). Each curve represents the average kinetics derived from 15 independent measurements of F_t_. **a** Normalized between F_O_ and F_M_: V_t_ = (F_t_ – F_O_)/(F_M_ – F_O_), and △V_t_ was marked by the O, L, K, J, I, P steps. The graph was plotted on a logarithmic time scale (0.02 ms to 1 s). **b** Normalized between F_O_ and F_K_: W_ok_ = (F_t_ – Fo)/(F_K_ – Fo), and △W_Ok_ reveals L-bands which indicate the degree of energetic dis-connectivity (grouping) of the PSII units. The graph was plotted on a linear time scale (0.02 ms to 0.3 ms). **c** Normalized between F_O_ and F_J_: W_oJ_ = (F_t_ – F_O_)/(F_J_ – F_O_), and △W_OJ_ reveals K-bands which indicate the degree of inactivation of OEC. The graph was plotted on a linear time scale (0.02 ms to 2 ms). **d** In the main panel, normalized between F_I_ and F_P_: W_IP_ = (F_t_ – F_I_)/(F_P_ – F_I_), and horizontal dashed line at 0.5 indicates half time needed to reduce pool of the end electron acceptor with electrons donated by intermediate carriers. In the inserted panel, normalized between F_O_ and F_I_: W_OI_ (≥1) = (F_t_ – F_O_)/(F_I_ – F_O_), and the maximum amplitude of IP phase illustrates the differences in the pool size of the end electron acceptors. The graph was plotted on a linear time scale (30 ms to 400 ms)

Various additional normalizations and difference kinetics were employed to reveal bands that were hidden in F_t_ (Fig. 4). F_t_ was firstly normalized between the step O and P and presented as relative variable fluorescence, V_t_ (Fig. 4a). The O, L, K, J, I and P steps were marked in the plot. Positive ΔL-, ΔK-, ΔJ- and ΔI-bands were displayed in ΔV_t_ (Fig. 4a), and the most distinct peak appeared at K step in mid-veins. Compared to leaf laminae, mid-veins had positive L-bands (Fig. 4b) and K-bands (Fig. 4c), as well as less half time and the maximum amplitude of IP rise (Fig. 4d).

**Fig. 5:**
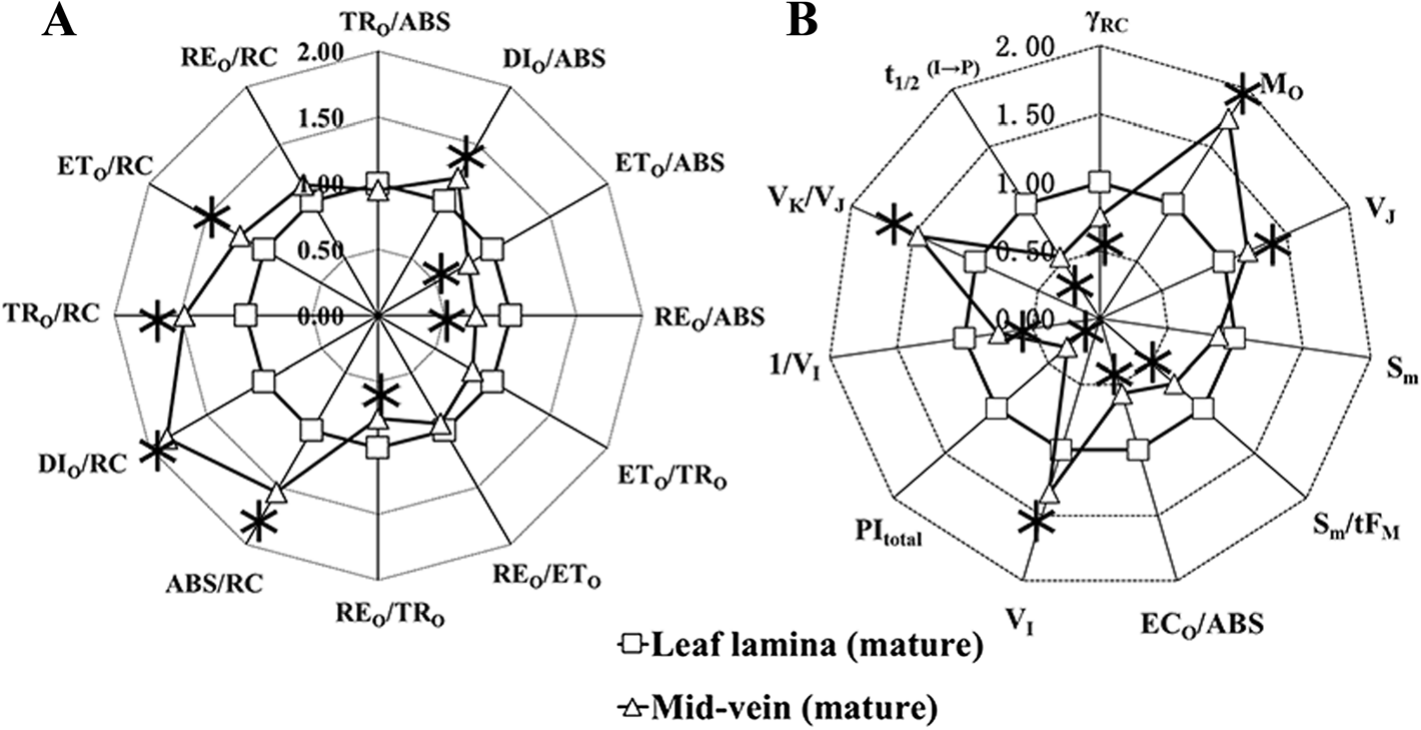
Radar plot representation of the behavior of structural and functional parameters. **a** Quantum yields, flux ratios, and specific energy fluxes per absorption flux (ABS) and reaction center (RC), Mid-veins (△) and leaf laminae (□). **b** Performance indexes, density of RCs and other fluorescence parameters. Each parameter was derived from JIP-test of the corresponding F_t_, and then normalized to the leaf laminae (with value of 100 % = 1). The significant difference between two tissues (*P* < 0.05) is indicated by the asterisk. The parameters represent the average kinetics collected from 15 independent measurements

To provide further quantitative and precise information, radar plot graphs were presented in Fig. 5a, b. The behavior of structural and functional parameters was analyzed according to the JIP-test (Strasser and Srivastava 1995; Strasser et al. 2004). The parameter definition and derivation were shown in Additional file 1: Table S1. Although specific fluxes parameters (ABS/RC, TR_O_/RC, ET_O_/RC and RE_O_/RC), V_K_/V_J_, V_J_, M_O_ and the parameters about heat dissipation (DI_O_/ABS and DI_O_/RC) increased in mid-veins, S_m_/t_FM_, γ_RC_, 1/V_I_, t_1*/*2_^(I−P)^, ET_O_/TR_O_, ET_O_/ABS, EC_O_/ABS, RE_O_/ABS, RE_O_/TR_O_ and PI_total_ in mid-veins exhibited a smaller value than those in leaf laminae. Other parameters, such as S_m_, TR_O_/ABS and RE_O_/ET_O_ did not differ significantly between two tissues.

**Fig. 6:**
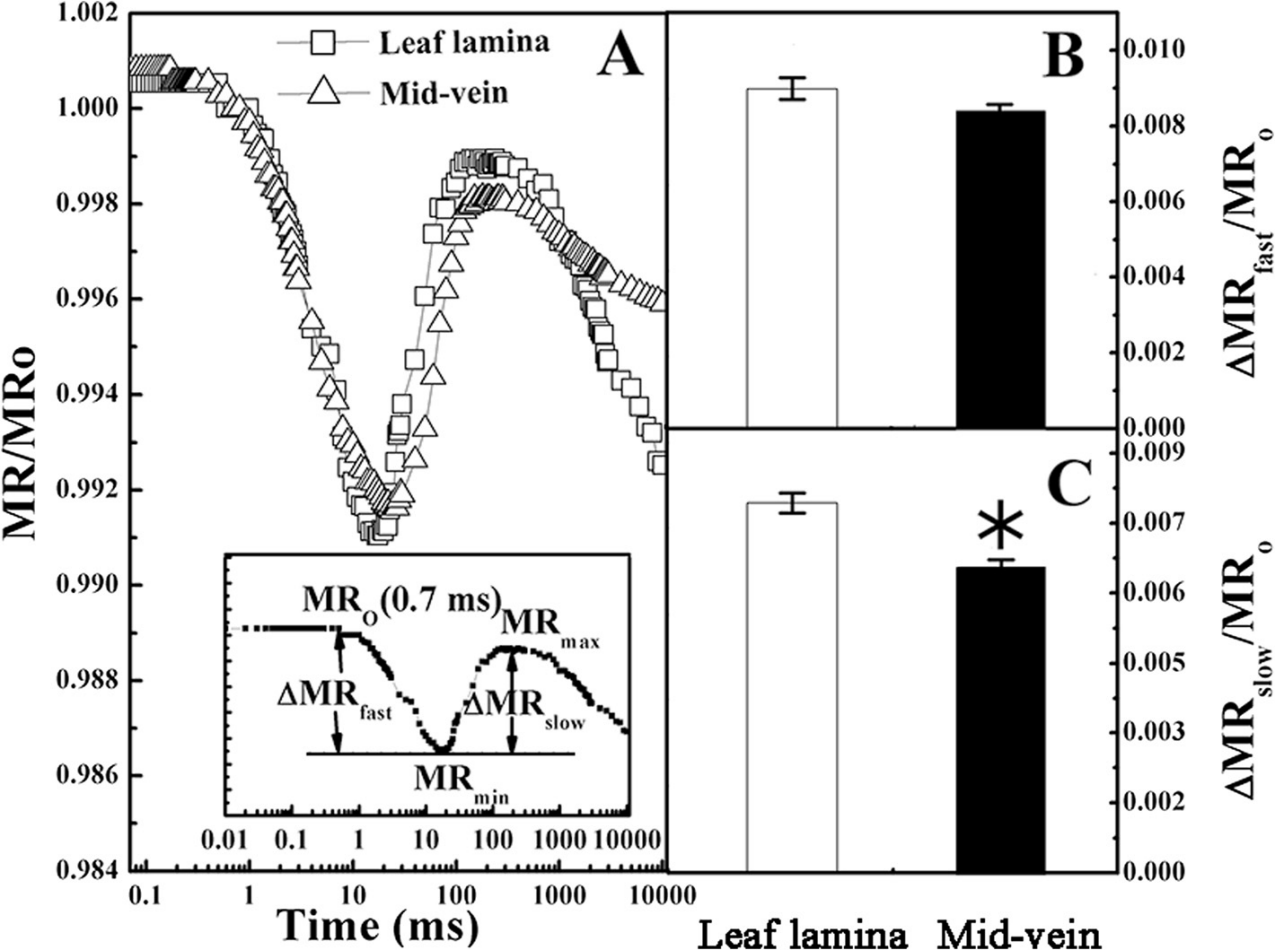
Mean kinetics of modulated reflection at 820 nm (MR). **a** MR was normalized and expressed by MR/MR_O_ on logarithmic time scale from 0 ms to 10 s, where MR_O_ was the value at 0.7 ms (taken at the onset of the red light illumination, the first reliable MR measurement). Each curve represents the average kinetics recorded from 15 independent measurements. The inserted graph showed the definition of characteristic parameters of the MR/MR_O_ kinetics (MR_min_, MR_max_) according to Strasser et al. (2010), where MR_min_ was the minimal MR reached during the fast phase (i.e., between 0.7 ms and 10–200 ms) and MR_max_ was the maximal MR reached by the end of the slow phase (taken at 1 s). **b** Amplitudes of the fast phase: ΔMR_fast_/MR_O_ = (MR_O_ − MR_min_)/MR_O_, which corresponds to the kinetics of the photo-induced changes in P700 redox state and the activated PSI RC (P700^+^). Mid-veins (△) and leaf laminae (□). **c** Amplitudes of the slow phase: ΔMR_slow_/MR_O_ = (MR_max_ − MR_min_)/MR_O_, which reflects P700^+^ re-reduction and connectivity of the two photosystems. Data are means ± SE from 15 independent measurements. The significant difference between two tissues (*P* < 0.05) is indicated by the asterisk

To test PSI activity and the connectivity of the two photosystems, the kinetics of the normalized modulated reflection at 820 nm (MR/MR_O_) were recorded according to Strasser et al. (2010). As shown in Fig. 6a, the amplitude of MR/MR_O_ diminished in mid-veins as compared to leaf laminae. To further characterize MR/MR_O_, the derived parameters (△MR_fast_/MR_O_ and △MR_slow_/MR_O_) from MR/MR_O_ were shown in Fig. 6b and c. The fast phase (△MR_fast_/MR_O_) had no significant difference in both tissues, whereas the slow phase (△MR_slow_/MR_O_) was distinctly higher in leaf laminae than mid-veins.

### Rice mid-veins possessed a lower linear electron flow

**Fig. 7:**
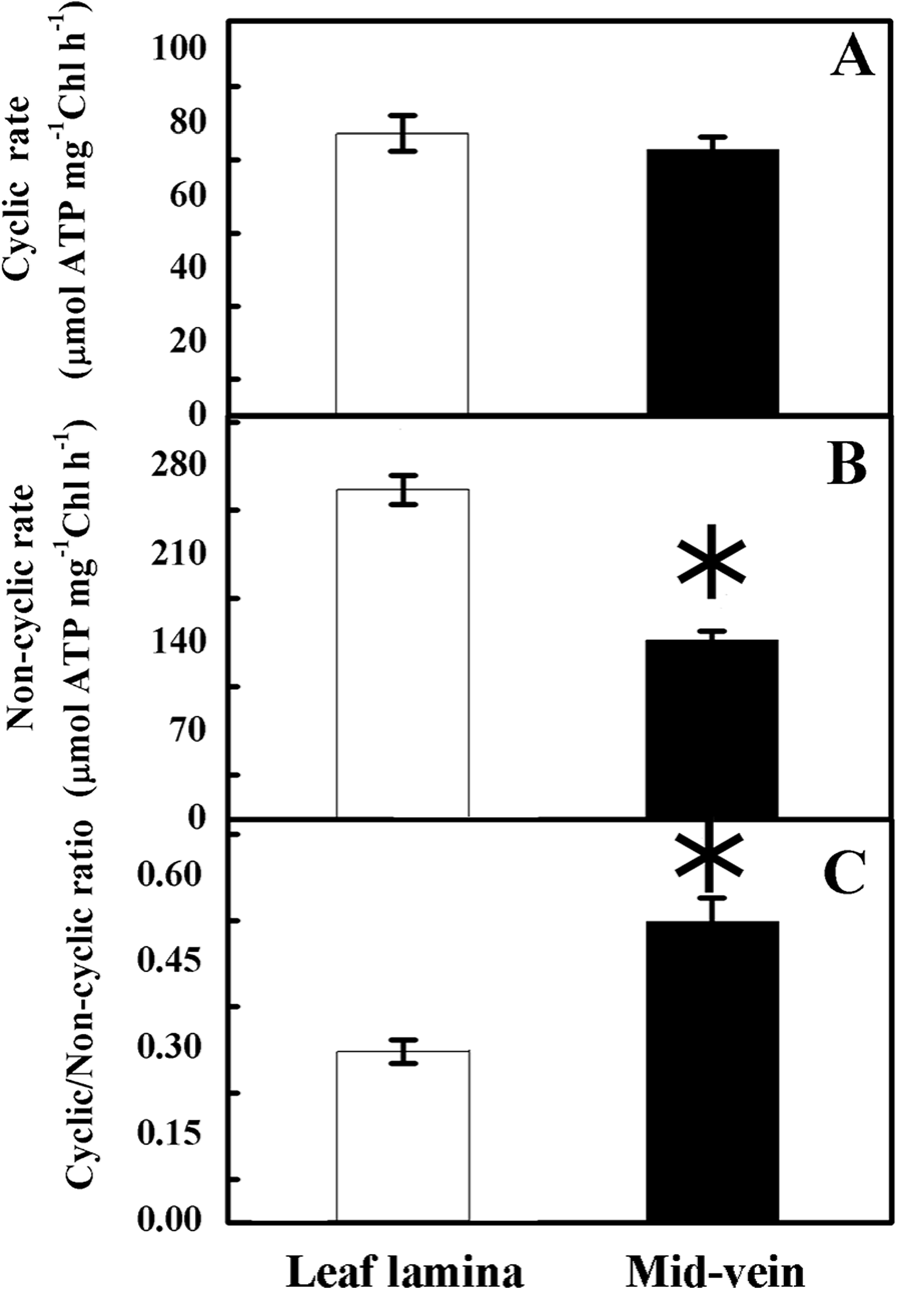
Cyclic (**a**), non-cyclic/linear (**b**) photophosphorylation activity, and their ratios (**c**) of mid-veins and leaf laminae. Data are means ± SE from 5 independent measurements. The significant difference between two tissues (*P* < 0.05) is indicated by asterisk

To further confirm that mid-veins had an unbalanced requirement for the electron transport flows, the cyclic and non-cyclic (linear) photophosphorylation rate of the chloroplasts were measured. Non-cyclic phosphorylation rate of mid-veins was lower than that of leaf laminae, while cyclic photophosphorylation exhibited no obvious changes (Fig. 7a and b). Hence, mid-veins were superior in cyclic/non-cyclic photophosphorylation ratio (Fig. 7c).

### Rice mid-veins had a lower accumulation of PSII supercomplexes and selective subunits

**Fig. 8:**
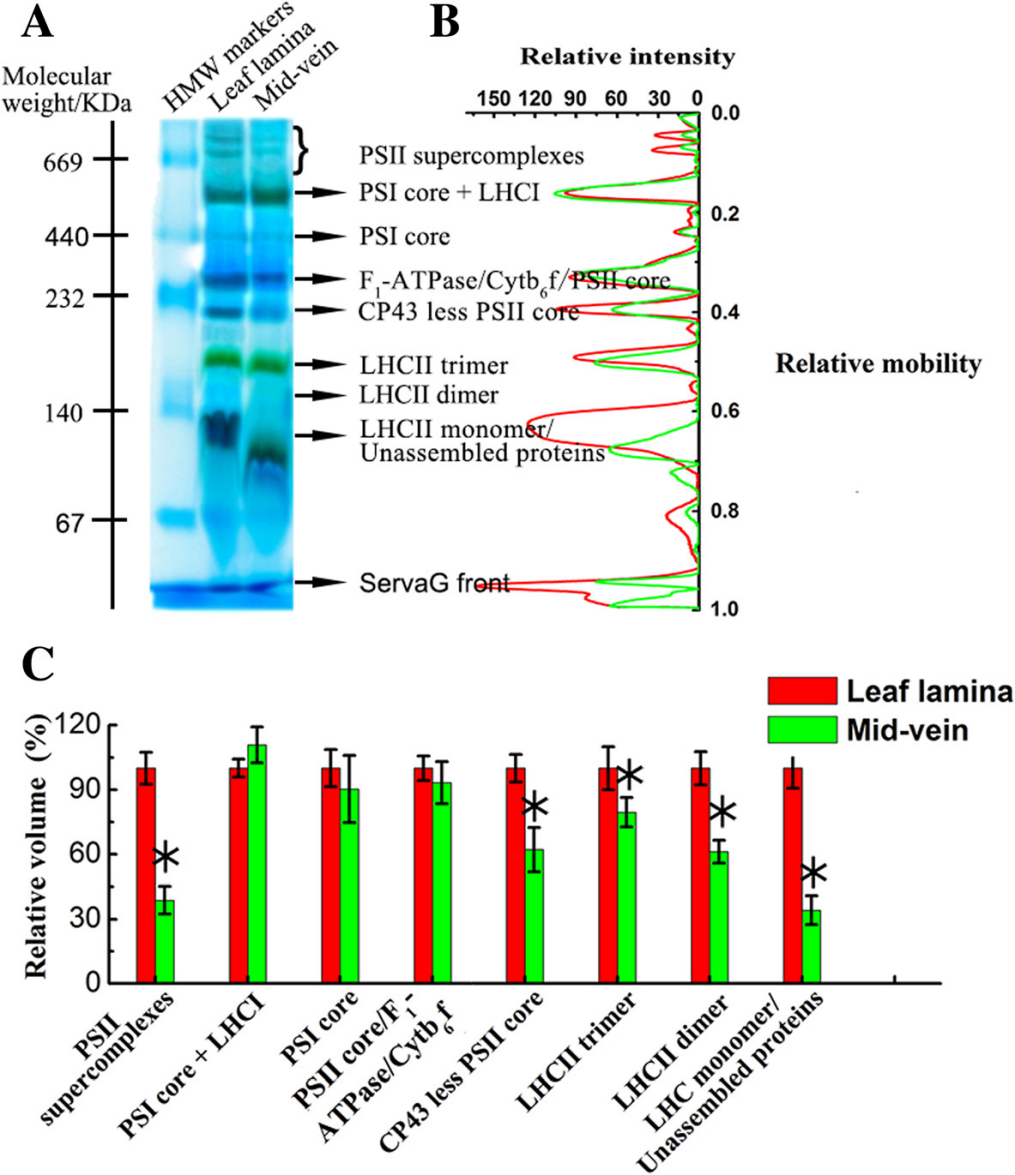
BN-PAGE profile of thylakoid membranes isolated from rice mid-veins (green) and leaf laminae (red). **a** Protein complexes (10 mg Chl per spot) were subjected to BN-PAGE electrophoresis. Molecular weight of HMW markers was labeled on the left, and the nomenclature of the resolved bands was labeled on the right. **b** Quantitation of the complexes. The BN-gels were scanned and the optical density along each lane was quantified (Quantity one, Bio-Rad, USA) to determine the relative mobility. **c** The abundance of each complex band. The abundance of each band was calculated by integrating the peak areas, and expressed as the percentage of that in leaf laminae

Thylakoid multi-subunit complexes can be separated in their native form with high resolution in Blue native polyacrylamide gel electrophoresis (BN-PAGE), whereas specific subunit stoichiometry of these complexes can be detected with immunoblots (Takabayashi et al. 2009). To understand the molecular mechanism for the modified photosystems in mid-veins, the assembly status of thylakoid membrane complexes was determined by BN-PAGE (Fig. 8a). Seven major bands were resolved, apparently corresponding to PSII supercomplexes, PSI-light harvesting complex (LHC) I, PSI core, ATP synthase-Cytb_6_f-PSII core, CP43 less PSII core, LHCII trimer, and dimer.

As compared to leaf laminae, we did not observe any obvious differences for PSI core + LHCI, PSI core, and ATP synthase-Cytb_6_f complex-PSII core, whereas PSII supercomplexes and CP43 less PSII core in mid-veins were far below detection in the leaf laminae. LHCII trimer and LHCII dimer were also reduced in the mid-vein (Fig. 8b and c).

**Fig. 9:**
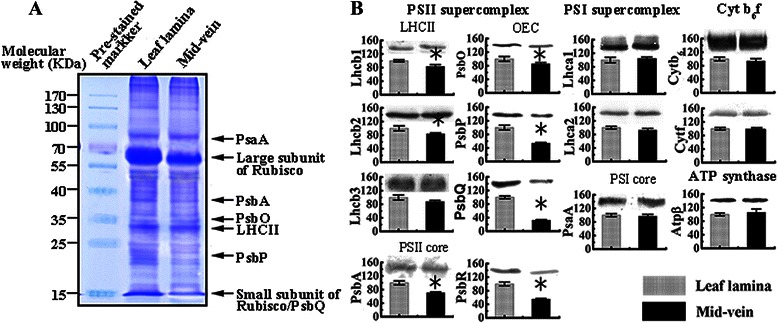
Immunoblot analysis of photosynthetic subunits in rice mid-veins and leaf laminae. **a** For protein loading (15 μg per spot) verification, SDS–PAGE gels were stained with Coomassie brilliant blue R-250. Pre-stained protein marker was indicated on the left, and bands corresponding to PsaA, large subunit of RuBisCO, PsbA, PsbO, LHCII, PsbP, and small subunit of RuBisCO/PsbQ were shown on the right. **b** Immunoblot analysis was carried out using the polyclonal antibodies against the thylakoid membrane subunits. Quantitation of band signal was performed using Quantity One (Bio-Rad, Hercules, USA) based on densitometric analysis. The amount of protein was expressed as the percentage of that in leaf laminae (100 %). Data are means ± SE from three independent experiments, with the asterisk indicating statistically significant differences (*P* < 0.05) between mid-veins and leaf laminae

In order to fingerprint the composition of these complexes, representative subunits were examined by immunoblots (Fig. 9). In PSII supercomplexes, the level of PsbP, PsbQ of OEC and PsbR were reduced most significantly than other subunits. PsbO of OEC, PsbA (PSII core subunit), Lhcb1 and Lhcb2 (LHCII subunits) were also decreased in mid-veins. Lhcb3 level was not altered significantly. In contrast, the relative level of the Cytb_6_f subunits, Cyt b_6_ and Cyt f, and ATP synthase subunits, Atpβ were not vulnerable in rice mid-veins. In respect of PSI supercomplex, PsaA (PSI core subunits), Lhca1 and Lhca2 (LHCI subunits) were also not changed in mid-veins (Fig. 9b).

## Discussion

### Rice mid-veins are photosynthetically active with high enrichment of C_4_ acid decarboxylases

With transverse section of rice leaves under epifluorescence or optical microscopy, both mid-veins and leaf laminae were proved to be photosynthesizable with chloroplasts (Fig. 1b, c). The chlorophyllous cells also border the vascular system in dicots such as celery, tobacco (Hibberd and Quick 2002), and woody species (Berveiller and Damesin 2008). Therefore, the cells around the veins in C_3_ plants (PCSVS) can also be termed ‘BS’ cells as C_4_ plants (Kinsman and Pyke 1998; Hibberd and Quick 2002).

The stems and petioles of tobacco (Hibberd and Quick 2002) and mid-veins of *Arabidopsis thaliana* (Brown et al. 2010) possess high activities of decarboxylating enzymes (NADP-ME, NAD-ME and PEPCK) and PPDK. Thus the ability of PCSVS to decarboxylate organic acids is phylogenetically widespread among C_3_ dicotyledons (Aubry et al. 2011). Our results firstly provided direct molecular evidence to the accumulation of decarboxylating enzymes in monocot rice mid-veins by immunoblot analyses (Fig. 2). The preferential accumulation of decarboxylating enzymes in rice mid-veins is analogous with that in BS cells of C_4_ plants beyond the typical range observed for C_3_ leaf laminae (Marshall et al. 2007; Kocurek and Pilarski 2011). Therefore, the location of chlorophyllous cells near PCSVS could be advantageous in terms of the carbon assimilation, which allow the decarboxylation of malate from the xylem and phloem, thus releasing CO_2_ for C_3_ cycle.

Indeed, these enzymes recruited into C_4_ photosynthesis fulfill conserved roles in distantly related C_3_ plants. During C_3_ plant defense response, PEPCK provides PEP to the shikimate pathway for the biosynthesis of aromatic compounds (Leegood et al. 1999; Lai et al. 2002). PPDK increases in rice roots during anoxia (Moons et al. 1998). NADP-ME2 also appears to be involved in the generation of reactive oxygen species (Voll et al. 2012). Especially, those members abundant in the PCSVS play an indispensable role in C_3_ plants. For example, in *Arabidopsis*, cucumber, and grape, PEPCK is suggested to be localized in phloem companion cells, where it may function in nitrogenous metabolism and pH regulation (Walker et al. 1999; Delgado-Alvarado et al. 2007; Malone et al. 2007); NAD-ME, NADP-ME and PPDK would also be linked with vascular bundles of C_3_ plants. NADP-ME and NAD-ME operate in the decarboxylation direction to supply CO_2_ to photosynthesis in mid-veins, and regulate the flux of carbon into soluble sugars, amino acids and glucosamine (Hibberd and Quick 2002; Brown et al. 2010).

Meanwhile, our results also found lower activities (Fig. 2a) and accumulations (Fig. 2b, c) of RuBisCO in rice mid-veins. It is consistent with the observation in *Arabidopsis* grown under elevated CO_2_ conditions (Bae and Sicher 2004), and in BS cells of NADP-ME type C_4_ plants. It is proposed that the elevated CO_2_ reduces the requirement for low-CO_2_-affinity RuBisCO in mid-veins.

### Rice mid-veins are equipped with C_4_-like photosystems, with lower linear electron transport

Leaf laminae chloroplasts of C_3_ plants function primarily in linear electron transport, which produces 3 ATP and 2 NADPH per O_2_ evolved to meet the requirements for C_3_ cycle (Finazzi et al. 2002). In contrast, in the NADP-ME type C_4_ plants, the decarboxylation of malate in BS cells results in a donation of NADPH. Therefore, Chl a fluorescence results from BS cells of C_4_ maize show the closure of PSII reaction center (RC) and a low PSII activity (Ivanov et al. 2005). BS cells lack PSII-initiated linear electron transport and only function to generate ATP by PSI-mediated cyclic electron flow. The ATP production per NADPH is about 2-fold higher in BS chloroplasts than that in M chloroplasts (Voznesenskaya et al. 1999).

Higher activity of three C_4_ acid decarboxylases also allows PCSVS in C_3_ mid-veins to decarboxylate malate for generating NADPH (Hibberd and Quick 2002). Hence, mid-veins similarly encounter the particular metabolic demands of a higher ATP/NADPH ratio as BS cells of C_4_ plants (Kotakis et al. 2006; Kalachanis and Manetas 2010). Since the demands would further shape the structure and function of photosystems and the associated electron flow (Kalachanis and Manetas 2010), we decided to further evaluate the differences of photosystems between mid-veins and leaf laminae by the non-invasive spectral technology.

V_t_ from F_t_ was higher in mid-veins than leaf laminae (Fig. 4a), indicating that the fraction of closed PSII RCs is higher at any time (Kalachanis and Manetas 2010; Yiotis and Manetas 2010) in rice mid-veins. The significantly increased initial slope of the fluorescence transient (M_O_) and decreased average redox state of Q_A_^−^/Q_A_ in the time span from 0 to t_FM_ (S_m_/t_FM_) (Fig. 5b) in mid-veins also demonstrate that mid-veins have a higher proportion of closed RCs of PSII (Chen and Cheng 2009). The positive value of L-bands indicates a low energetic connectivity/grouping of PSII units (Strasser et al. 2004). Hence, the higher L-bands in mid-veins (Fig. 4b) further suggest that the closed RCs of PSII lose stability and become inefficient to transfer energy.

ΔV_t_ (Fig. 4a) uncovered the main bottleneck of PSII occurring at K step. The appearance of positive K-bands in mid-veins (Fig. 4c) reflects an inactivation of the OEC at the donor side of PSII (Yusuf et al. 2010). The destruction of OEC, also supported by higher V_K_/V_J_ quantitation (Fig. 5b) and a decrease in ΔMR_slow_/MR_O_ (Oukarroum et al. 2012), would allow light to over-energize PSII and deactivate PSII RCs. Meanwhile, the lower γ_RC_ (the fraction of PSII Chl a molecules that function as RCs), and higher ABS/RC (a relative measure of antenna size feeding active RCs) (Fig. 5) in mid-veins suggest that the harvesting antenna complexes (LHC) of PSII were relatively larger than active RCs (Kirst et al. 2012). Therefore, higher proportions of absorbed energy in mid-veins needs to be dissipated as heat, which was supported by the higher DIo/RC (dissipated energy flux per RC) and DI_O_/ABS (quantum yield for energy dissipation, Fig. 5a). An inactivated fraction of RCs also forced each RC to bear more specific fluxes of absorbed, trapped energy, electron transfer and reduction of PSI end electron acceptors (ABS/RC, TR_O_/RC, ET_O_/RC and RE_O_/RC, Fig. 5a) in mid-veins.

IP phase depends on electron transfer through PSI and is caused by the transient block at the acceptor side of PSI (NADP^+^) (Schansker et al. 2005). The maximal amplitude of IP phase, parameterized by 1/V_I_, represents the relative pool size of the final electron acceptors of PSI (Yusuf et al. 2010). As NADP^+^ is continuously consumed by C_4_ acid decarboxylases, mid-veins are supposed to have a smaller pool of PSI end electron acceptors with the lower maximal amplitude of IP (Fig. 4d) and 1/V_I_ (Fig. 5b).

On the other hand, the operation of cyclic electron flow in C_4_ BS chloroplasts is also studied by the redox kinetics of P700^+^, which predominantly excites PSI. Since the electron flow solely from PSII is insufficient to reduce the active PSI RCs, the decarboxylation of malate generates NADPH as an electron donor to PSI for a larger cyclic electron flow (Ivanov et al. 2005). Our results similarly showed that the activation status of PSI RC (P700^+^), as indicated by ΔMR_fast_/MR_O_ (Fig. 6b), in rice mid-veins was kept as high as in leaf laminae. Consequently, filling of PSI acceptors with electrons proceeded faster in mid-veins (Fig. 4d), resulting in the shorter time needed for half saturation of these pools with electrons donated by intermediate carriers (t_1*/*2_^(I−P)^, Fig. 5b). This phenomenon is also observed in pericarps of *Nerium oleander* where the completion of electron flow toward the acceptor side of PSI is likely facilitated due to the reduced final electron acceptor pools of PSI and their high affinities for electrons (Kalachanis and Manetas 2010). Therefore, the quantum yield for reduction of end electron acceptors at the PSI acceptor side (RE_O_/ABS), and efficiency/probability that a trapped excitation can move an electron into the electron transport chain from Q_A_- to the PSI end electron acceptors (RE_O_/TR_O_) were lower in mid-veins (Fig. 5a). Better-preserved pools of intermediate carriers (S_m_, Fig. 5b) and activation status of PSI RC (P700^+^) (ΔMR_fast_/MR_O_, Fig. 6c) further ensured mid-veins to achieve higher cyclic electron flow (Fig. 7a) by diverting electrons back to intermediate carriers around PSI (Kalachanis and Manetas 2010), while the maximal performance for linear electron transport from water to reduce the end electron acceptor, PI_total_ (Fig. 5b), was still precluded with lower efficiency/probability that an electron comes into the linear electron transport chain beyond Q_A_^−^ of PSII RCs (ET_O_/TR_O_ and ET_O_/ABS accordingly, Fig. 5a).

### Rice mid-veins lacking linear electron flow is attributed to a selective loss of PSII-bound subunits

In C_4_ plants, M thylakoids have a complete linear electron transport chain, containing PSII, Cytb_6_f, and PSI complexes, similar to C_3_ leaf laminae. In contrast, BS thylakoids contain fewer functional PSII, but normal number of PSI, Cytb_6_f and ATP synthase complexes which primarily participate in cyclic electron transport. Subunits of PSI, LHCI, Cyt b_6_f, and ATP synthase complexes show BS/M accumulation ratios of 1.6, 1.72, 1.0, and 1.33, respectively, whereas ratios for the PSII and LHCII were 0.45 and 0.68, respectively (Wojciech et al. 2008).

As direct molecular evidence for the physiological data, BN-PAGE and immunoblot analysis of the thylakoid photosynthetic apparatus prove an uneven distribution of photosystems between mid-veins and leaf laminae in rice. Either the integrated forms or CP43-less core of PSII supercomplexes was far below detections in leaf laminae (Fig. 8). The loss of PSII complexes explains the dysfunction of PSII and the hindered linear electron transport rates in mid-veins as mention above. Considering the subsets of PSII supercomplexes, the accumulation of LHCII trimers and their subunits (Lhcb1 and Lhcb2) was reduced less than PsbA (PSII core subunit) in rice mid-veins (Fig. 9b). It corresponds well to the fluorescence results that LHCII is larger than active PSII RCs.

Early studies in NADP-ME type C_4_ species maize (*Zea may*) and sorghum (*Sorghum bicolor*) show that limited PSII activities in chloroplasts of BS cells are mainly caused by the depletion in PsbP and PsbQ of OEC and PsbR, which play an important role in water oxidation (Meierhoff and Westhoff 1993). Likewise, the loss of PsbP, PsbQ and PsbR in mid-veins was more than other PSII subunits (Fig. 9b), although not as significant as that in BS cells of C_4_ plants (Meierhoff and Westhoff 1993). Hence, K-bands (inactivation of OEC, Fig. 4c) in rice mid-veins also perhaps result from the selective loss of distinct OEC polypeptides (PsbP and PsbQ). An RNAi-induced *Arabidopsis* mutant lacking detectable PsbP proteins exhibits a significant defect in electron transfer from Q_A_^−^ to Q_B_ with loss of the J to I transition of F_t_, and shows seriously retarded charge recombination between Q_A_^−^ and OEC (Andréasson et al. 1995). Therefore, removals of PsbP and PsbQ of OEC are speculated to be the most important reasons for introducing linear electron transport defects in the PSII.

Furthermore, rice mid-veins assembled intact PSI core complex (Fig. 8), PsaA (PSI core subunit), and Lhca1 and Lhca2 (LHCI subunits, Fig. 9b) into the oligomeric forms of PSI-LHCI (Fig. 8). Cyt b_6_f and ATP synthase complexes (Fig. 8), as well as their subunits (Cytf, Cytb_6_ and Atpβ, Fig. 9b) were also well-preserved in rice mid-veins. If most of the excitation energy is utilized by PSI, cyclic electron transport around PSI and Cytb_6_f may prevail over linear electron flow mediated by both PSII and PSI (Finazzi et al. 2002). Therefore, the particular photosystems in mid-veins ensured the cyclic electron flow to work efficiently at the expense of the linear one (Fig. 7).

C_4_ photosynthesis is considered as one of the most convergent of the complex evolutionary phenomena on Earth, and the majority of C_4_ crop species have quite a degree of mechanistic flexibility and circumstantial superiority (Furbank 2011). Introducing their multigenic trait into rice has been recognized as an ambitious and multinational project for increasing rice yields. Without doubt, it still faces enormous challenges, because it is not clear how C_4_ photosynthesis has evolved independently from the ancestral C_3_ pathway (Sage et al. 2011), and how the metabolism of the rice leaf will be changed after introduction (Aubry et al. 2011). However, our finding that the existence of C_4_-like photosynthesis (high enrichment of C_4_ acid decarboxylases, lower linear electron transport and a selective loss of PSII-bound subunits) in rice mid-veins makes it seem more feasible to introduce components of the C_4_ pathway into rice and gives some indication that the evolution of C_4_ photosynthesis may not be as difficult as first appears (Kajala et al. 2011).

## Conclusions

In summary, this was the first study showing higher levels of C_4_ cycle key enzymes and special adjustments in the photosynthetic machinery in rice mid-veins. The linear electron transport chain was mostly blocked by a “traffic jam” occurring on PSII complexes, whereas PSI in mid-veins is sufficient to support a larger cyclic one. The loss-of-function of PSII was primarily attributed to selective subunits, such as PsbP and PsbQ of OEC and PsbR. These attributes of photosynthetic machinery in rice mid-veins partly resemble that in the BS cells of C_4_ plants, as mentioned in Introduction. Hence, our findings indicate that the photosynthetic cells surrounding mid-veins in the rice, a typical C_3_ monocot, innately possess C_4_-like features as do other C_3_ dicots. Of course, further research is needed to extend these findings to more C_3_ plants. Such studies would provide a simple explanation for the polyphyletic evolution of C_4_ photosynthesis (Hibberd and Quick 2002).

## Methods

### Plant materials and growth conditions

*Oryza sativa*. cv. Liangyoupeijiu, a typical monocot C_3_ plant was pot-cultivated, watered and fertilized routinely in a net house under natural conditions at the Institute of Agricultural Sciences of Jiangsu Nanjing, China (32° 03’ N, 118° 47’ E). Air temperature, rainfall and global radiation were available in previous studies (Yu et al. 2012). The flag leaves of the main culm were sampled during mature stage (24 days after leaf emergence). To avoid regional discrepancy due to developmental stages, only midsections of flag leaves were utilized. For in vitro experiments, mid-veins were pulled out from leaf laminae according to a method developed by Brown et al. (2010), and both mid-veins and residual leaf laminae were immediately frozen in liquid nitrogen, and stored at −80 °C. Several plant samples were pooled to obtain sufficient material for further analyses. For fast Chl a fluorescence transients and modulated 820 nm reflection experiments in vivo, leaf laminae and mid-veins were still attached to the rice, and the measurements were operated at ambient temperature and 8–10 a.m.

### Determination of chlorophyllous cell distribution

Transverse sections of leaves containing mid-veins and leaf laminae were cut manually using razor blades, and mixed with a glycerol-PBS solution (50 % glycerol, 137 mM NaCl, 2.7 mM KCl, 10 mM Na_2_HPO_4_, and 2 mM KH_2_PO_4_, pH = 7.2) for optical and epifluorescence microscopy (Nikon CFI60, Tokyo, Japan). Chlorophyllous cells were visualized as bright red fluorescence (LP 520) when excited with blue light (BP 450–490) in epifluorescence microscopy.

### Enzyme-linked assays

Enzymes were isolated according to Ivanov et al. (2006). Briefly, samples were ground in liquid nitrogen and extracted with 1 ml of a buffer containing 100 mM Tris/HCl (pH 7.6), 5 % (w/v) PVP-40, 0.85 % (w/v) BSA, and 10 mM DTT.

The enzyme-linked assays for detection of PEPCK (EC 4.1.1.32), NAD-ME (EC 1.1.1.39), NADP-ME (EC 1.1.1.40) and PPDK (EC 2.7.9.1) were conducted as described previously (Ashton et al. 1990; Marshall et al. 2007). Enzymatic activities of PEPC (EC 4.1.1.31) and RuBisCO (EC 4.1.1.39) were determined according to Tietz and Wild (1991) and Berveiller and Damesin (2007), respectively. Enzyme activity was expressed as μmol substrate consumed or product generated per second to total Chl content. Experiments were carried out at 25 °C.

### Measurement of fast Chl a fluorescence transients (JIP curve, F_t_) and modulated 820 nm reflection (MR)

Light intensity actually falling on a cylindrical mid-vein cannot be accurately determined and small sized mid-veins cannot fill the leaf clip space. Therefore, photon exchange between the instrument and mid-veins or leaf laminae was through a 3 mm × 15 mm rectangular window. The window was a non-fluorescing black tape aligned along the mid-vein axes which was made by ourselves according to the method of Manetas (2004). Leaf laminae and mid-veins, from the midsection of the same leaf and still attached to plants, were mounted in a leaf clip fitted by the window and dark adapted for 60 min before measurements.

F_t_ was captured by a Handy Plant Efficiency Analyzer (Hansatech Instruments Ltd, Norfolk, UK) according to Strasser and Srivastava (1995) after excitation by a band of three red light emitting diodes (650 nm, 3000 μmol photons m^−2^ s^−1^). Data were recorded from 20 μs to 1 s, and analyzed according to the JIP test (Strasser et al. 2004; Jiang et al. 2008) for calculation of structural and functional parameters. The corresponding definitions and calculations of the parameters were given in Additional file 1: Table S1. The average values were expressed as ratios to that of leaf laminae in radar plots. Extended analysis of F_t_ was done by normalization as various relative variable fluorescence (V or W) between different time points, according to previous studies (Strasser et al. 2007; Tsimilli-Michael and Strasser 2008). The difference fluorescence kinetics (ΔV or ΔW) between mid-veins and leaf laminae were calculated through the equation: ΔV(W) = V(W)_mid ‐ vein_ – V(W)_leaf lamina_. See details below: (A) normalized between F_O_ and F_M_: V_t_ = (F_t_ – F_O_)/(F_M_ – F_O_), and △V_t_ was marked by the O, L, K, J, I, P steps. The graph was plotted on a logarithmic time scale (0.02 ms to 1 s). (B) normalized between F_O_ and F_K_: W_ok_ = (F_t_ – Fo)/(F_K_ – Fo), and △W_Ok_ revealed L-bands which indicated the degree of energetic dis-connectivity (grouping) of the PSII units. The graph was plotted on a linear time scale (0.02 ms to 0.3 ms). (C) normalized between F_O_ and F_J_: W_oJ_ = (F_t_ – F_O_)/(F_J_ – F_O_), and △W_OJ_ revealed K-bands which indicated the degree of inactivation of OEC. The graph was plotted on a linear time scale (0.02 ms to 2 ms). (D) normalized between F_I_ and F_P_: W_IP_ = (F_t_ – F_I_)/(F_P_ – F_I_), and horizontal dashed line at 0.5 indicated half time needed to reduce pool of the end electron acceptor with electrons donated by intermediate carriers. (E) normalized between F_O_ and F_I_: W_OI_ (≥1) = (F_t_ – F_O_)/(F_I_ – F_O_), and the maximum amplitude of IP phase illustrated the differences in the pool size of the end electron acceptors of PSI. The graph was plotted on a linear time scale (30 ms to 400 ms).

Modulated reflection at 820 nm (MR) was recorded on a Multifunctional Plant Efficiency Analyzer M-PEA (Hansatech Instrument Ltd., Norfolk, UK), according to an operating protocol elucidated by Strasser et al. (2010). MR was normalized and expressed by MR/MR_O_ on logarithmic time scale from 0 ms to 10 s, where MR_O_ was the value at 0.7 ms (taken at the onset of the red light illumination, the first reliable MR measurement). The definition of characteristic parameters of MR/MR_O_ kinetics (MR_min_ and MR_max_) was shown on an inserted graph (Fig. 6a), where MR_min_ represented the minimal MR reached during the fast phase (i.e., between 0.7 ms and 10–200 ms) and MR_max_ was the maximal MR reached by the end of the slow phase (taken at 1 s). Each curve represented the average kinetics collected from 15 independent measurements of 15 individual plants and was used to calculate relevant parameters.

### Isolation of thylakoid membrane and total soluble protein

Thylakoid membrane was isolated from mid-veins and leaf laminae as described earlier (Kang et al. 2012). The Chl content was determined spectrophotometrically in 80 % acetone (Arnon 1949). Total soluble proteins were isolated as described by Ku et al. (1999). PPDK proteins needed to be concentrated before immunoblot (Chastain et al. 2002). Protein concentration was determined using the method of Lowry et al. (1951), with bovine serum albumin as a standard.

### Immunodetection of photosynthetic proteins

The isolated thylakoid membrane (for thylakoid membrane subunits) and total soluble protein (for key enzymes in C_3_ or C_4_ cycles) were pretreated with the loading buffer (0.5 M Tris–HCl, pH 6.8, 1 % SDS, 24 % glycerol, 4 % β-mercaptoethanol, and 0.001 % w/v bromophenol blue) and denatured for 10 min at 90 °C. Thylakoid membrane polypeptides (2 μg Chl per spot) or total soluble protein (15 μg protein per spot) from mid-veins and leaf laminae were separated by 12 % SDS-PAGE. After electrophoresis, gels were stained with Coomassie brilliant blue R-250 or transferred electrophoretically to PVDF membranes (0.2 μm pore size, Bio-Rad, USA). PVDF membranes were subsequently incubated with primary antibodies raised in rabbits against key enzymes in C_3_ or C_4_ cycles [the large subunit of RuBisCO, PEPC, PPDK, PEPCK (Agrisera, Sweden, http://www.agrisera.com/), NAD-ME and NADP-ME (Beijing Protein Innovation, China, http://proteomics.bioon.com.cn/)] and the thylakoid membrane subunits [Lhcb1, Lhcb2, Lhcb3, PsbA, PsbO, PsbP, PsbQ, PsbR, Lhca1, Lhca2, PsaA, Cytb_6_, Cytf, Atpβ (Agrisera, Sweden, http://www.agrisera.com/)] at recommended dilutions. Afterward, samples were incubated in goat anti-rabbit IgG conjugated with alkaline phosphatase (Bio-Rad, USA). The immunoblot signals were visualized using BCIP/NBT (Roche, Switzerland) as substrate according to Lindahl et al. (1996).

### Blue native polyacrylamide gel electrophoresis (BN-PAGE)

For the separation of thylakoid membrane complexes, BN-PAGE was carried out in the procedure described by Chen et al. (2007) with some modifications. Briefly, the thylakoid membranes were washed with 330 mM sorbitol and 50 mM BisTris-HCl (pH 7.0), and then resuspended in buffer containing 20 % glycerol, 25 mM BisTris-HCl (pH 7.0) at 1.0 mg Chl ml^−1^. The suspension was solubilized with an equal volume of resuspension buffer containing 2 % (w/v) dodecyl-β-D-maltoside. After incubation at 4 °C for 30 min, insoluble material was removed by centrifugation at 40,000 × g for 10 min. The supernatant equal to 10 mg Chl was mixed with one-tenth volume of 1 % Coomassie brilliant blue G-250 in 100 mM BisTris-HCl (pH 7.0), 0.5 M 6-amino-n-caproic acid, and 30 % glycerol. The mixture was then applied to 1-mm-thick 5–12 % polyacrylamide gradient gels for electrophoresis. Electrophoresis was performed at 4 °C, 120 V for 5 h. High molecular weight native protein marker kit (GE Healthcare, Amersham-Pharmacia, 17-0445-01, UK) was used to determine the molecular mass of these complexes.

Quantitation of band signal in BN-PAGE and immunoblot analyses was performed using Quantity One, version: 4.52 (Bio-Rad, Hercules, USA) based on densitometric analysis. The experiments were repeated three times and the representative images were taken. The amount of protein was expressed as the percentage of that in leaf laminae.

### Chloroplast isolation and cyclic/non-cyclic (linear) photophosphorylation rate assays

Chloroplast isolation was performed according to Ketcham et al. (1984). The cyclic/non-cyclic (linear) photophosphorylation activity of chloroplasts was assessed by using the luciferin-luciferase method to measure the amount of ATP synthesized within 2 min at a saturating irradiance of about 1,500 μmol quanta m^−2^ s^−1^ at 25 °C (Allnutt et al. 1991).

### Statistical analyses

Statistical analyses were carried out using SPSS 15.00 statistical package (Chicago, USA). Parametric one-way ANOVA was used to determine statistical significance between leaf laminae and mid-veins. Differences in the measured parameters were considered significant at *p* < 0.05.

## Additional file

Additional file 1: Table S1. Formulae and definitions of the selected JIP-test fluorescence parameters used in this study. (DOCX 38 kb)

## Abbreviations

ABS: absorption flux
BN-PAGE: blue native polyacrylamide gel electrophoresis
BS: bundle sheath
Chl: chlorophyll
DI_O_: dissipated energy flux
EC_O_: total electron carriers
ET_O_: electron transport flux
F_t_: fast chlorophyll fluorescence kinetics
M: mesophyll
MR: modulated reflection at 820 nm
NAD-ME: nicotinamide adenine dinucleotide-dependent malic enzyme
NADPH: the reduced form of nicotinamide adenine dinucleotide phosphate
NADP-ME: nicotinamide adenine dinucleotide phosphate-dependent malic enzyme
OEC: oxygen evolving complex
PCSVS: photosynthetic cells surrounding the vascular system
PEPC: phosphoenolpyruvate carboxylase
PEPCK: phosphoenolpyruvate carboxykinase
PPDK: pyruvate phosphate dikinase
PSI: photosystem I
PSII: photosystem II
RC: reaction center
RE_O_: reduction of end acceptors of PSI
RuBisCO: ribulose-1, 5-bisphosphate carboxylase/oxygenase
TR_O_: trapped energy flux
