# Supplementary material for: The existence of C4-bundle-sheath-like photosynthesis in the mid-vein of C3 rice
Source: Rice (N Y). 2016 May 10;9:20. doi: 10.1186/s12284-016-0094-5 (PMC4864733; doi:10.1186/s12284-016-0094-5)
Supplement: Additional file 1: Table S1. — Formulae and definitions of the selected JIP-test fluorescence parameters used in this study. (DOCX 38 kb) [file 12284_2016_94_MOESM1_ESM.docx]

**Additional file 1**

**Table S1. Formulae and definitions of the selected JIP-test fluorescence parameters used in this study.**

| **Fluorescence parameter** | **Definition** |
| --- | --- |
| **Original data extracted from the recorded fluorescence transient F_t_** | |
| F_t_ | Fluorescence at time t after onset of actinic illumination |
| F_0_ ≌ F_20µs_ | Minimal reliable recorded fluorescence at 20 µs with the Handy-PEA-fluorimeter, when all PSII RCs are open |
| F_P_ = F_M_ | Maximum fluorescence, when all PSII RCs are closed |
| F_L_ ≡ F_150µs_ | Fluorescence intensity at the L-step (about 150 µs) |
| F_k_ ≡ F_300µs_ | Fluorescence intensity at the K-step (300 µs) |
| F_J_ ≡ F_2ms_ | Fluorescence intensity at the J-step (2 ms) |
| F_I_ ≡ F_30ms_ | Fluorescence intensity at the I-step (30 ms) |
| F_p_ (=F_M_) | Maximal recorded fluorescence intensity at the peak P of OJIP, when all PSII RCs are closed |
| t_FM_ | Time (in ms) to reach F_M_ |
| Area | Total complementary area between the fluorescence induction curve and F = F_M_ |
| **Fluorescence parameters derived from the original data** | |
| Selected OJIP parameters |  |
| V_t_ ≡ (F_t_ - F_0_)/(F_M_ - F_0_) | Relative variable fluorescence at time t |
| V_K_ = (F_K_ - F_0_)/(F_M_ - F_0_) | Relative variable fluorescence at the K-step |
| V_J_ = (F_J_ - F_0_)/(F_M_ - F_0_) | Relative variable fluorescence at the J-step |
| V_I_ = (F_I_ - F_0_)/(F_M_ - F_0_) | Relative variable fluorescence at the I-step |
| 1/V_I_ = (F_M_ - F_0_)/(F_I_ - F_0_) | The maximal amplitude of IP phase reflecting the relative pool size of the final electron acceptors of PSI |
| M_o_ = 4(F_300µs_ - F_0_)(F_M_ - F_0_) | Approximated initial slope (in ms^–1^) of the fluorescence transient normalized on the maximal variable fluorescence F_M_ - Fo |
| S_m_ = Area/(F_M_ - F_0_) = EC_0_/RC | Normalized total complementary area above the OJIP transient (reflecting multiple turnover Q_A_ reduction events) or total electron carriers per RC |
| EC_0_/ABS = (EC_0_/RC)(RC/ABS) | Electron carriers per ABS at t = 0 |
| V_K_/V_J_ | A relative measure of inactivation of OEC |
| t_1/2_^(I-P)^ | The time needed for half saturation of the final electron acceptors pools of PSI with electrons donated by intermediate carriers |
| Quantum yields or flux ratios | |
| φ_Po_ = TR_0_/ABS = 1 - F_0_/F_M_ | Maximum quantum yield for primary photochemistry at t = 0 |
| φ_Eo_ = ET_0_/ABS = (1 - F_0_/F_M_)(1 - V_J_) | Quantum yield for electron transport at t = 0 |
| φ_Ro_ = RE_0_/ABS = φ_Po_ψ_Eo_δ_Ro_ | Quantum yield for reduction of end electron acceptors at the PSI acceptor side at t = 0 |
| φ_Do_ = DI_O_/ABS = 1 - φ_Po_ = F_0_/F_M_ | Quantum yield for energy dissipation at t = 0 |
| ψ_Eo_ = ET_0_/TR_0_ = 1 - V_J_ | Efficiency/probability with which a trapped excition can move an electron into the electron transport chain beyond Q_A_^-^ |
| δ_Ro_ = RE_0_/ET_0_ = (1 - V_I_)(1 - V_J_) | Efficiency/probability with which an electron can move from the reduced intersystem electron acceptors to the PSI end electron acceptors |
| ρ_Ro_ = RE_0_/TR_0_ = ψE_0_δR_0_ | Efficiency/probability with which a trapped excition can move an electron into the electron transport chain from Q_A_^-^ to the PSI end electron acceptors |
| Specific energy fluxes (per Q_A_-reducing PSII RC) | |
| ABS/RC = M_0_/V_J_/φ_Po_ | Absorption flux per RC (reflecting an average antenna size) |
| TR_0_/RC = M_0_/V_J_ | Trapped energy flux (leading to Q_A_ reduction) per RC at t = 0 |
| ET_0_/RC = M_0_(1/V_J_)(1 - V_J_) | Electron transport flux (further than Q_A_) per RC at t = 0 |
| RE_0_/RC = M_0_(1/V_J_)ψ_Eo_δ_Ro_ | Electron flux reducing end electron acceptors at the PSI acceptor side per RC at t = 0 |
| DI_0_/RC = (ABS/RC) - (TR_0_/RC) | Dissipated energy flux per RC at t = 0 |
| Density of RCs | |
| γ_RC_ = Chl_RC_/Chl_total_ = RC/(ABS + RC) = V_J_φ_Po_/( M_0_ + V_J_φ_Po_) | Probability that a PSII Chl molecule functions as RCs |
| S_m_/t_FM_ = [RC_open_/(RC_close_ + RC_open_)]av = [Q_A_/Q_A(total)_]av | Average fraction of open RCs (redox state of Q_A_^-^/Q_A_) of PSII in the time span between 0 and t_FM_ |
| Performance indexes (products of terms expressing partial potentials at steps of energy bifurcations) | |
| PI_total_ = (RC/ABS)[φ_Po_/(1-φ_Po_)] [ψ_Eo_/(1-ψ_Eo_)][δ_Ro_/(1-δ_Ro_)] | Performance index (potential) for energy conservation from exciton to the reduction of PSI end acceptors |

Subscript “0” (or “o” when written after another subscript) indicates that the parameter refers to the onset of illumination, when all RCs are assumed to be open.
